# Editorial: Fertility preservation in the pediatric and adolescent populations, volume II

**DOI:** 10.3389/fendo.2024.1372225

**Published:** 2024-02-01

**Authors:** Mahmoud Salama, Yasmin Jayasinghe

**Affiliations:** ^1^ Department of Obstetrics, Gynecology and Reproductive Biology, Michigan State University, East Lansing, MI, United States; ^2^ Department of Obstetrics and Gynecology, University of Melbourne, Royal Women’s Hospital, Parkville, VIC, Australia; ^3^ Oncofertility Program, Royal Children’s Hospital, Melbourne, VIC, Australia; ^4^ Murdoch Children’s Research Institute, Melbourne, VIC, Australia

**Keywords:** oncofertility, cancer, fertility preservation, pediatric, adolescent

The Frontiers in Endocrinology Research Topic on fertility preservation (FP) in the pediatric and adolescent populations invited authors from across the globe to participate in the dissemination of knowledge and awareness regarding the best fertility preservation principles in the pediatric and adolescent populations.

This Research Topic serves as a dedicated research publication, or part thereof, to highlight the important aspects of missing research information in pediatric and adolescent fertility preservation programs and aims to advance the science considerably. Since many centers, nationally and internationally, are not even aware of best practice guidelines for pediatric and adolescent fertility preservation in children and adolescents facing fertility threatening diagnoses and treatment plans, disseminating knowledge regarding the same in both providers and thus the population at large, is much needed.

The importance of FP is being increasingly recognized, with many international guidelines now removing the lower age limit for offering FP (1–4). This Research Topic demonstrates the significant knowledge gains in oncofertility that continue to be achieved in the young population.

A review by Chen et al. demonstrates the continuing progress in fertility preservation technologies. Ovarian tissue cryopreservation is deemed innovative, however continues to advance with over 200 births estimated to have been achieved by 2020 (5), with average live birth rates around 23% (6). Consecutive pregnancies (the highest being four) from the same graft have been seen, making it a very efficient fertility preservation method (7). Research efforts to protect against malignant contamination after tissue grafting are ongoing. Recurrence of malignancy has been reported in 3.9-7%, and none thought to be related to the ovarian tissue grafting process (8, 9).

Recent cases of successful prepubertal oocyte collection have been reported (in a 7-year-old patient with Turner’s mosaicism who collected 6 oocytes (10), and a transgender male under 12 years who collected 9 oocytes) (11) raising questions about best fertility preservation options for young birth-assigned female patients. This is addressed further in the first systematic review of oocyte collection in 468 females and transgender males ≤ 18 years (median age 15.2 years) by Slonim et al.. The majority of stimulation cycles (96.3%) successfully obtained oocytes and complications were rare. The highest success was seen in the transgender population (compared to those about to receive gonadotoxic therapy or those with Turner syndrome). Only one live birth from cryopreserved oocytes has been reported in this age group (12), with the authors recommending that oocyte collection in post-pubertal adolescents be regarded as innovative, while for prepubertal patients it should be considered experimental due to unknown oocyte quality.

For birth-assigned males, over 1000 testicular tissue biopsies were reported by 2020 (13). However successful human birth after testicular tissue cryopreservation has not been seen. Following on from the success of the first primate birth in 2019 (14), Younis et al. describe *in vitro* maturation of immature testicular tissue from both pre and peripubertal males to primary spermatocyte stage after being maintained in organotypic culture for 32 days. While complete spermatogenesis was not seen, these findings provide increasing hope that by the time a prepubertal male reaches adulthood, realistic attempts at parenthood may be made.

Populations who can potentially benefit from fertility preservation procedures continue to expand. In this Research Topic, Rodriguez-Wallberg et al. reported on outcomes of 100 women with Turner syndrome and recommend referral for fertility preservation counselling after onset of puberty to maximize yield. Follicles were seen in 25% of ovarian tissue biopsies analyzed and were more likely present in adolescents compared to prepubertal children or adults. Similarly, reports on quality of ovarian and testicular biopsies in those with rare diagnoses such as mucopolysaccharidoses and Diamond Blackfan syndrome and disorders of sexual differentiation (frameshift mutations) are reported by Ruan et al. and Teoli et al. respectively, which can further help clinicians with fertility preservation counselling for these patients.

One of the most complex aspects of fertility preservation in children is the triadic nature of decision making (between the parent, patient and the clinician). The clinician plays an important role assessing comorbidities, mitigating risk and communicating clear and unbiased medical recommendations to families in order to support informed decision-making. Parents who are the surrogate decision makers for younger children may experience conflict and concern around making choices that might be incongruent with the child’s future wishes (15). In this new Frontiers Research Topic Takae et al. report findings from a novel study examining comprehension and attitudes of children and adolescents towards fertility preservation before and after age-appropriate explanation, tailored to the understanding of the individual child. This included the use of storytelling for very young children. The majority of participants were females with median age of 14 (6-17) years. Before the intervention, the majority of children under 11 years (63.4%) did not know if they wanted children in the future and were non-committal about learning about fertility preservation options, but this changed after education, with over 90% wanting to hear more about fertility preservation options. While children should not be relied upon to take responsibility or consent to fertility preservation procedures, for those who are submature and have some understanding, it is appropriate to have their agreement or at least not have their disagreement prior to having a surgical intervention.

Finally, implementation of fertility preservation programs and cryobanks is another priority in order to address disparities in care (16). In this Research Topic Baston-Büst et al. discuss their step-wise approach for implementing a cryobank in Germany in a university-based setting, including engagement of key-stakeholders and development of standard operating procedures for ovarian tissue cryopreservation (**Figure 1**). Quality assurance and audit is a regulation in most laboratories but should also be part of any clinical fertility preservation program through the development of national oncofertility registries to enable collaboration and meaningful evaluation of long-term outcomes of fertility preservation interventions. Accordingly, Ozimek et al. concluded that although oncofertility services are expanding globally, very few countries have well-established official national oncofertility registries. The authors highlight the urgent need for having a well-established official national oncofertility registry in each country to monitor oncofertility services in a way that best serves patients.

**Figure 1:**
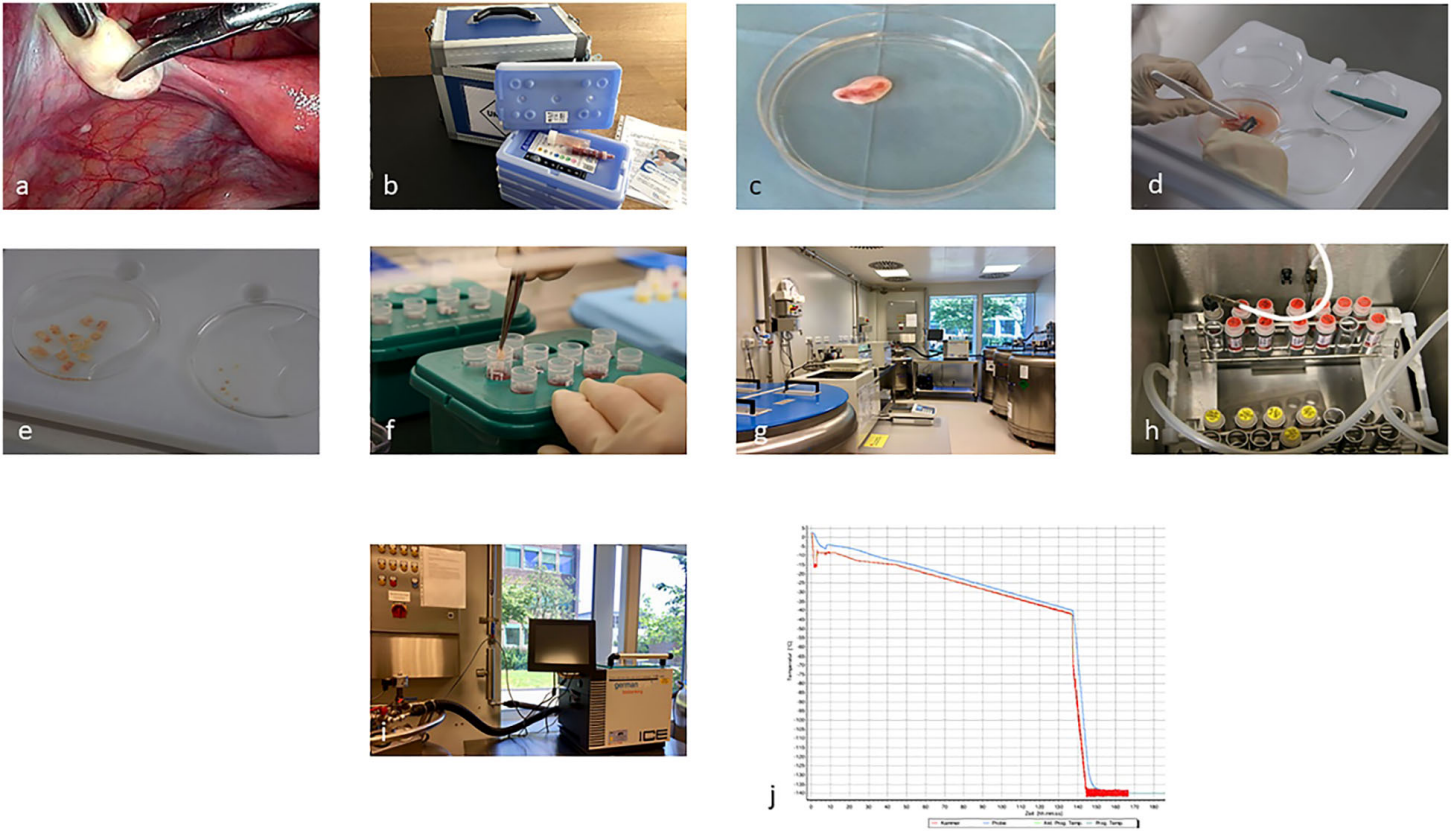
Ovarian tissue cryopreservation process from laparoscopic biopsy **(A)**, (overnight) transport **(B)**, to preparation and freezing of the cortical biopsies **(C–J)** (Baston-Büst et al.).

## Author contributions

MS: Writing – original draft, Writing – review & editing. YJ: Writing – original draft, Writing – review & editing.
